# Cutaneous melanoma: descriptive epidemiological study

**DOI:** 10.1590/S1516-31802008000100008

**Published:** 2008-01-03

**Authors:** Nelson Marcos Ferrari, Helena Muller, Manoel Ribeiro, Marcus Maia, José Antonio Sanches

**Keywords:** Melanoma, Epidemiology, Sex distribution, Limbs, Survival rate, Melanoma, Epidemiologia, Distribuição por sexo, Extremidades, Taxa de sobrevida

## Abstract

**CONTEXT AND OBJECTIVE:**

Cutaneous melanoma represents around 3% of all skin tumors. About 20% of such patients will have advanced disease and will die before reaching five years of survival. The aim of this paper was to describe the clinical and histopathological variables and their correlations.

**DESIGN AND SETTING:**

Retrospective, descriptive, epidemiological study at the Melanoma Unit, Dermatological Clinic, Irmandade da Santa Casa de Misericórdia, São Paulo.

**METHODS:**

Records from 364 cases between May 1993 and January 2006 were analyzed. The frequencies of all study variables and their 95% confidence intervals were determined. The chi-squared test was used to evaluate associations among the variables, adopting a significant level of 0.05.

**RESULTS:**

Females predominated, with 1.4 women for each man. The patients’ mean age was 58.9 years. Nonwhite patients represented 13.7% of the sample. The prevalent anatomical sites for cutaneous melanoma were the trunk and feet, for both men and women. Acral lentiginous melanoma represented 22.3% of the cohort. *In situ* primary lesions were observed in few cases and a high percentage of thick cutaneous melanoma was detected. Ulceration was found in 13.4% of the thin tumors (≤ 1.0 mm). Thicker and ulcerated lesions predominated in male patients (p = 0.011 and p < 0.001 respectively) and in elderly patients (p = 0.021 and p = 0.015).

**CONCLUSIONS:**

The cohort mostly presented thick and ulcerated tumors, denoting late diagnosis and bad prognosis. Also, the sample was characterized by considerable prevalence of female patients, nonwhite patients, limb lesions and acral lentiginous melanoma.

## RESUMO

**CONTEXTO E OBJETIVO:**

O melanoma cutâneo representa cerca de 3% de todos os tumores da pele. Em torno de 20% dos pacientes terão doença avançada e morrerão antes de completar cinco anos de sobrevida. O objetivo deste artigo é descrever as variáveis clínicas e histopatológicas, e suas correlações.

**TIPO DE ESTUDO E LOCAL:**

Estudo retrospectivo, epidemiológico, descritivo realizado na Unidade de Melanoma, Clínica de Dermatologia, Irmandade da Santa Casa de Misericórdia de São Paulo, Brasil.

**MÉTODOS:**

364 casos foram estudados de maio de 1993 a janeiro de 2006. Utilizou-se o intervalo de confiança de 95% para as análises e para as associações o teste do qui-quadrado bicaudal. Estabeleceu-se nível de significância de 0,05.

**RESULTADOS:**

Predominou o sexo feminino resultando em uma proporção de 1,4 mulheres para cada homem. A média das idades dos pacientes foi de 58,9 anos. Para homens e mulheres, o melanoma cutâneo localizou-se, predominantemente no tronco e pés. O melanoma acrolentiginoso representou 22,3% de toda amostra. Observou-se minoria de casos com lesão primária *in situ* e alto percentual de melanoma cutâneo espesso. Ulceração foi observada em 13,4% para tumores finos (≤ 1,0 mm). Homens apresentaram lesões mais espessas (p = 0,011) e ulceradas (p < 0,001) e também idosos (p = 0,021 para a espessura e p = 0,015 para ulceração).

**CONCLUSÕES:**

Esta casuística constitui-se, na sua maioria, de tumores espessos e ulcerados, o que significa diagnóstico tardio e pior prognóstico. Também se caracterizou por presença marcante de sexo feminino, pacientes não-brancos, tumores em extremidades e melanoma acrolentiginoso.

## INTRODUCTION

During the second half of the twentieth century, the incidence of skin cancers increased, along with the resultant mortality. The reason for these increases is probably related to changes in people's habits, including types of leisure and clothes. Cutaneous melanoma (CM) only accounts for 3% of all skin tumors. However, CM presents significant morbidity and a high mortality rate, accounting for 75% of all deaths due to cutaneous malignant neoplasms and 1 to 2% of all cancer deaths in the United States, thus representing a veritable public health problem.^1^

Differently to other types of cancer, CM frequently affects younger individuals, with a mean age of fifty years. For this reason, CM is among the most serious cancers when evaluated according to number of years of life lost. Moreover, despite all the efforts of therapies used in advanced cases, such as chemotherapy, radiotherapy, biochemical therapy and vaccines, these seem to be incapable of providing cures or improving survival rates.

Knowledge of the epidemiological, clinical and histological features of CM may contribute towards better comprehension of the disease. This could facilitate medical actions aimed at identifying cases, as well as helping in planning campaigns among the medical community and the population. From a search in the literature, we were unable to find any epidemiological study that presented a bigger sample than ours.

## OBJECTIVES

The present study had the aim of analyzing the clinical and histopathological data on patients with cutaneous melanoma, taking into considering the primary lesion site, growth pattern, Breslow thickness, Clark level, presence of ulceration and stage of the melanoma. Demographic variables like the patients’ gender, age, and skin color were also evaluated. Finally, correlations between the variables were determined.

## METHODS

Between May 1993 and January 2006, 364 CM patients were registered, treated and followed up in the Melanoma Unit of Hospital Irmandade da Santa Casa de Misericórdia de São Paulo, São Paulo, Brazil.

In order to evaluate the variables, the melanoma was staged in accordance with the guidelines of the American Joint Committee on Cancer (AJCC)^2^ of 2002.

Descriptive age statistics were calculated, taking into consideration only the cases belonging to the group with localized disease, since all of them presented an initial diagnosis that was precisely determined. In addition, the patients were divided into two groups: up to 65 years and over 65 years.

Considering the skin color, the patients were divided into two groups: whites and nonwhites. The latter group included black, medium-brown and yellow patients. The presence of ulceration was recorded based on the anatomopathological examination.

In the statistical analysis, frequencies and 95% confidence intervals of the variables were determined. Following this, the chi-squared test was applied to investigate any associations between the variables, taking a significance level of 0.05.

## RESULTS

The study group was composed of 214 females and 150 males, thus giving a ratio of 1.4 women per man. The mean age was 58.9 years and the median was 61.0 years. CM was predominantly found in patients aged 41-60 years (33.6%) and 61-80 years (45.6%) (Table 1).

**Table 1.** Distribution of the cutaneous melanoma cases, according to gender, age, skin color, primary lesion site and growth pattern

Gender	Frequency	%
Female	214	58.8
Male	150	41.2
**Total**	**364**	**100.0**
**Age**			
≤ 20	1	0.4
21 – 40	35	14.0
41 – 60	84	33.6
61 – 80	114	45.6
> 80	16	6.4
**Total**	**250**	**100.0**
**Skin Color**			
White	314	86.3
Nonwhite	50	13.7
**Total**	**364**	**100.0**
**Lesion site**			
Limbs	178	50.6
Trunk	105	29.8
Head and neck	69	19.6
No primary lesion	12	3.2
**Total**	**364**	**100.0**
**Growth pattern**			
Superficial spreading melanoma	123	33.8
Nodular melanoma	95	26.1
Acral-lentiginous melanoma	81	22.3
Lentigo maligna melanoma.	42	11.5
Others	23	6.3
**Total**	**364**	**100.0**

The sample consisted of 314 white (86.3%), 34 black (9.3%), 14 medium brown (3.8%) and two East Asian (0.5%) patients. The lesions were located on the limbs (178), trunk (105) and head and neck (69). Twelve patients did not present any primary lesions.

Superficial spreading melanoma (SSM) was found in 123 cases (33.8%), nodular melanoma (NM) in 95 cases (26.1%), acral-lentiginous melanoma (ALM) in 81 cases (22.3%) and lentigo maligna melanoma (LMM) in 42 cases (11.5%). Other types of melanoma were found in 23 cases (6.3%) (Table 1).

The mean thickness of the primary tumors was 4.88 mm and the median was 2.5 mm. In 20 cases (5.6%) it was not possible to obtain such data. The distribution of the cases according to Breslow thickness ranges is demonstrated in Table 2. There was predominance of cases with Clark III (23.4%) and IV (25.4%) levels. Ulceration was found in 47.4% of the invasive tumors (Table 2).

**Table 2.** Distribution of the cutaneous melanoma cases, according to Breslow thickness, Clark level and presence of ulceration

Breslow thickness	Frequency	%
*In situ*	52	15.7
≤ 1.0 mm	67	23.9
1.01 to 2.0 mm	48	14.5
2.01 to 4.0 mm	52	15.7
> 4.0 mm	113	34.0
**Total**	**332**	**100.0**
**Clark level**			
I	52	15.6
II	49	14.7
III	78	23.4
IV	85	25.4
V	68	20.4
**Total**	**332**	**100.0**
**Ulceration**			
Yes	128	47.4
No	142	52.6
**Total**	**270**	**100.0**

The women predominantly presented tumors on their limbs, while the men had tumors on their limbs and trunk (Table 3). The gender distribution according to Breslow thickness ranges is shown in Table 4. Among the men, 52% of the lesions were ulcerated, while women presented 33.3% (p < 0.001) (Table 3).

**Table 3.** Distribution of the cutaneous melanoma cases by gender, in relation to the primary lesion site, Breslow thickness and presence of ulceration

Lesion site	Female		Male	
	Frequency	%	Frequency	%
Head and neck	42	20.0	27	19.0
Trunk	51	24.3	54	38.0
Arms	23	10.9	12	8.4
Legs	35	16.6	13	9.1
Hands	12	5.7	2	1.4
Feet	45	21.4	34	23.9
**Total**	**210**	**100.0**	**142**	**100.0**
**Breslow thickness**				
≤ 1.0 mm	49	42.1	15	26.2
1.01 to 2.0 mm	30	18.0	17	15.7
2.01 to 4.0 mm	26	27.7	27	33.8
> 4.0 mm	61	30.2	49	40.0
**Total**	**166**	**100.0**	**108**	**100.0**
**Ulceration**				
Yes	66	33.3	66	52.0
No	132	66.7	61	48.0
**Total**	**198**	**100.0**	**127**	**100.0**

p = 0.011.

p < 0.001.

**Table 4.** Distribution of the cutaneous melanoma cases by age, in relation to the growth pattern, Breslow thickness and presence of ulceration

Growth pattern	≤ 65 years		> 65 years	
	Frequency	%	Frequency	%
Superficial spreading melanoma	76	52.4	23	21.9
Nodular melaoma	34	23.4	19	18.1
Acral- lentiginous melanoma	22	15.2	33	31.4
Lentigo maligna melanoma	13	9.0	28	26.7
Others	_	_	2	1.9
**Total**	**145**	**100.0**	**105**	**100.0**
**Breslow Thickness**				
≤ 1.0 mm	40	33.8	17	22.3
1.01 to 2.0 mm	28	23.7	10	13.1
2.01 to 4.0 mm	20	16.9	16	21.0
> 4.0 mm	30	25.4	33	43.4
**Total**	**118**	**100.0**	**76**	**100.0**
**Ulceration**				
Yes	41	29.1	45	43.3
No	100	70.9	59	56.7
**Total**	**141**	**100.0**	**104**	**100.0**

p < 0.001.

p = 0.021.

p = 0.015.

Analysis of age in relation to the melanoma growth patterns showed predominance of the LMM and ALM types among elderly patients and SSM and MN patterns among non-elderly patients (Table 4). Ulceration was predominantly observed among patients older than 65 years of age (Table 4).

The CM tumors were predominantly on the limbs, and mainly on the feet (68.2%), among nonwhite patients (Table 5). There was predominance of the LMM, SSM and NM forms among white patients and the ALM form among nonwhite patients (Table 5).

**Table 5.** Distribution of the cutaneous melanoma cases by skin color, in relation to the lesion site and growth pattern

Lesion Site	White		Nonwhite	
	Frequency	%	Frequency	%
Head and neck	65	21.0	4	9.7
Trunk	100	32.3	5	12.1
Arms	35	11.3	–	–
Legs	44	14.2	4	9.7
Hands	14	4.5	–	–
Feet	51	16.5	28	68.2
**Total**	**309**	**100.0**	**41**	**100.0**
**Growth pattern**				
Superficial spreading melanoma	118	37.6	5	10.0
Nodular Melanoma	87	27.7	8	16.0
Acral-lentiginous melanoma	49	15.6	32	64.0
Lentigo maligna melanoma	40	12.7	2	4.0
Others	20	6.4	3	6.0
**Total**	**314**	**100.0**	**50**	**100.0**

p < 0.001.

The distribution of ALM cases showed that their primary lesions were predominantly on the plantar regions, for both white and nonwhite patients (Table 6).

**Table 6.** Distribution of 82 acral-lentiginous melanoma cases by skin color, in relation to the lesion site

Lesion Site	White		Nonwhite	
	n	%	n	%
Dorsum of hand	1	2.0	–	–
Subungual area of hands	5	10.2	4	12.1
Heel	5	10.2	4	12.1
Dorsum of foot	5	10.2	–	–
Subungual area of feet	6	12.2	1	3.1
Plantar region	27	55.2	24	72.7
**Total**	**49**	**100.0**	**33**	**100.0**

Concerning the staging of CM, the 52 cases (15.7%) of *in situ* tumors and 270 (84.2%) of invasive tumors were distributed into clinical stages (Table 7) and microstaging, in accordance with the AJCC, 2002 (Table 8).

**Table 7.** Distribution of 354 cases of cutaneous melanoma according to clinical staging

Staging	Number of cases	%
CS 0	52	14.6
CS I	89	25.1
CS II	106	29.9
CS III	74	20.9
CS IV	33	9.3
**Total**	**354**	**100.0**

CS = clinical stage.

**Table 8.** Distribution of 269 cases of invasive cutaneous melanoma according to tumor ulceration (presence/absence), in relation to the microstaging of the primary lesion

Staging	Ulceration	No ulceration	Total (%)
T1 (≤ 1.0 mm)	51	13	64 (23.9)
T2 (1.01 to 2.0 mm)	36	11	47 (17.4)
T3 (2.01 to 4.0 mm)	20	31	51 (18.9)
T4 (> 4.0 mm)	30	77	107 (39.7)
**Total**	**137**	**132**	**269 (100.0)**

With regard to the evolution of the 250 patients who initially presented localized disease, 53 (21.2%) evolved towards advanced melanoma stages, 38 (15.2%) to locoregional disease and 15 (6%) directly to systemic disease. Furthermore, 19 (50%) out of the 38 patients who had developed locoregional melanoma evolved to systemic CM, thus totaling 34 patients (13.6%) from the group with localized disease.

In the group of 74 patients with locoregional disease, 30 cases (40.5%) evolved to systemic disease. This number was added to the 19 cases in the group with localized CM, to total 49 cases (43.7%). The final number of cases presenting systemic CM was 97, which was the sum of the initial 33 cases, plus 15 from the group with localized disease, plus 49 from the locoregional group.

The mean survival of the patients with localized melanoma was 97.8 months (confidence interval, CI = 91.1 to 104.5) and the melanoma-specific survival rate was 85.1% over three years.

Considering all causes of death, the overall survival rate was 81% over three years, with a mean survival of 88.3 months (CI = 80.9 to 95.7). The follow-up period ranged from 1.1 months to 117.1 months. It was not possible to follow up 19.7% of the patients.

## DISCUSSION

CM is a potentially fatal malignant type of neoplasia with increasing incidence over the last few decades. The possibility of achieving a cure is conditioned to an early diagnosis. Knowledge of CM epidemiology is fundamental for both primary and secondary public health strategies.

Sixty percent of the subjects of this study were women, resulting in a ratio of 1.4:1. This predominance was observed in practically all age groups. Data in the international literature show that CM predominantly occurs among women in geographic regions with low sunlight indices and low CM incidence.^3^ In countries with high incidence of CM, like Australia, there is slight predominance of the male gender or a balance between genders.

The frequency of CM cases shown in the present study is similar to results from other Brazilian reports.^4-14^ In the international literature, data on incidence are usually cited with adjustments for gender, population and year. This impaired comparisons with our data. Curiously, English-language papers point towards increases in CM rates among men and declines among women in the near future.^15,16^ In Brazil, data for the year 2000 from the Brazilian Institute for Geography and Statistics (Instituto Brasileiro de Geografia e Estatística, IBGE) demonstrated a balanced ratio between men (49.2%) and women (50.8%).^17^

Since the Dermatological Clinic of the Santa Casa Hospital of São Paulo receives higher demand for medical attendance from women (56.8%), this could explain the predominance of women in this sample. Another possible explanation would be the constant exposure to the sun among Brazilians. Since Brazil is a tropical country, with high temperatures during a large part of the year, women wear clothes that would allow greater exposure to sunlight.

The majority of the patients in the present study, practically 80%, were in the age range of 41-80 years old (mean of 60 years old). The literature shows mean ages of between 50 and 58 years^18^ and increased incidence among elderly individuals, which will increase the mean age by a few years.^19,20^ This could be explained by lower solar exposure in infancy, since the population has more awareness of the risks of sunlight, thus diminishing the incidence of CM among young individuals. In Brazil, there is great variability in the data on age and mean age of CM onset, with values that are sometimes below or above the worldwide rates, such that it is difficult to establish a single standard.

CM usually predominates among Caucasian populations. Studying mixed populations, as in our sample, which presented a considerable percentage of nonwhite patients (13.7%), suggests the existence of other etiological factors associated with this disease.

The variability of ethnic components in any given population makes it difficult to classify individuals based on skin color, considering that medium-brown patients could be classified as either white or nonwhite. In Brazil, two types of population have been analyzed: one type in the southern region with predominance of white individuals, as found in samples studied in other parts of the world;^4,8,10,11,14,21^ and another type that occurs in the southeastern, central-western and northeastern regions, in which 10 to 20% of the sample are nonwhite individuals, as in our study.^5-7,9,12,13,22-24^

In our study, about 50% of the primary CM lesions were located on the limbs, while 30% were on trunk and 20% on the head. Both men (38%) and women (24.3%) displayed lesions predominantly on the trunk. Most Brazilian studies indicate predominance of lesions on the limbs (39-66%), without distinguishing whether they were on the hands or feet.^6-8,10,12-14,21-23,25^ We also found Brazilian reports showing predominance of lesions on the trunk among men and on the limbs among women.^8,9,11,21^ Recent international cohort studies have shown that the main lesion sites among white men are the trunk and head. Legs are rarely affected. Among white women, the legs and trunk are sites that are equally affected.^3^ Lesions on the limbs are more often detected among nonwhite individuals.

In our study, CM predominated on the trunk among white individuals (52.8%), as did the SSM (37.6%) and NM (27.7%) types, and on feet among non-white individuals (68.2%), of which 64% were of ALM type. These data are similar to those found in the literature.^26-29^ The ALM type predominated in the plantar regions of both nonwhite and white individuals. In the present study, the percentage of ALM found among white individuals (15.6%) was greater than in some reports (2-8%).^26,27^ One possible explanation could be the failure to distinguish white from nonwhite individuals.

Among women, the SSM type predominated (37.4%), while among men, the NM (32%) and the SSM (28.7%) types predominated. However, the frequency of ALM in this sample was high: about 25% for both genders.

Predominance of lesions on the head and neck (33.3%) and feet (27.7%) was shown among the elderly patients above 65 years old, and also the ALM (31.4%) and LMM (26.7%) melanoma types. The predominance of tumors located on the head and neck of elderly individuals can possibly be explained by the increased incidence of LMM at this age. It is also possible that, as a consequence of delayed ALM diagnosis, it could be more frequently detected at advanced ages. Individuals aged less than 65 years presented lesions located on the trunk (35.4%) and of ALM type (52.4%), predominantly.

The sample for this study presented peculiarities in relation to the site of the primary injury and the ALM frequency, probably because around 14% of the study population consisted of nonwhite patients. These data are comparable with national reports.^6,12,13,24^ Curiously, the predominance of CM located on limbs has also been found in populations from the southern region of Brazil, although their samples were composed of white patients.^8,10,14,21,25^

Concerning the melanoma thickness, the women presented a greater proportion (60%) of thinner CM (up to 2.0 mm) and the men presented a greater proportion (74%) of thicker CM (> 2.0 mm), which was in accordance with the literature.^15,16,30^ The women presented fewer ulcerated lesions. It is possible that women dedicate greater attention to their health and seek dermatological attendance more frequently. The thinnest CM (up to 2.0 mm) was of LMM and SSM types and the thickest (> 2.0 mm) was of NM and ALM types. This was expected, since the LMM and SSM types predominantly presented radial growth for longer periods of time. Of note is the fact that the ALM type is usually presented as a thick tumor at the time of the first attendance, possibly because of the patient's neglect or late diagnosis.

In our study, *in situ* neoplasia was in a minority (14.6%; clinical stage, CS) 0) but there was a high percentage of advanced cases (30.2%; CS III and CS IV). Around 40.0% of the primary tumors presented thickness > 4.0 mm and only 25% were ≤ 1 mm. Among the cases with thin CM, 25% had ulceration or Clark level IV or V, denoting a high percentage of cases with a bad prognosis. To validate the melanoma staging system devised by the AJCC, 17,600 patients from USA, Europe and Australia were analyzed. Only 10.0% were reported to present thick CM (> 4.0 mm), while 40% had thin CM (≤ 1 mm),^31^ i.e. the inverse of our study. Moreover, data from developed countries have demonstrated increased incidence of thin CM and stabilization of the incidence of thick CM.^3,16^ Almost half of the invasive CM among individuals with localized, locoregional and systemic disease in our study presented ulceration. Among cases of CM ≤ 1 mm, the percentage of ulceration was 13.4%. In the international literature, around 35% of the CM among patients with localized disease has been reported to be ulcerated and 6% of the thin CM is also ulcerated.^31^ A Brazilian study showed that 43.6% of the CM was ulcerated, without distinguishing whether it was thin or thick.^21^

The present sample is still small and has had insufficient follow-up for analyzing the survival curves relating to the study variables. However, it was found by calculating the CM-specific survival rate that 85.1% of the patients with localized disease were still alive after three years.

## CONCLUSIONS

Women predominated at practically all ages among our patients with CM, but without predominance of lesions located on the legs, contrary to the data found in the literature. The sample consisted of patients with predominance of thick and ulcerated tumors, allowing us to deduce that the CM was diagnosed late, with consequent worse prognosis. This was most evident among the elderly men.

Because of the ethnic composition, with a considerable number of nonwhite patients in the study population, a significant percentage of acral-lentiginous melanoma was observed. This predominance was responsible for the lower proportions of other types of melanoma, and therefore the frequency of superficial spreading melanoma cases was lower than what has been reported in the international literature and in most other Brazilian samples. A considerable percentage of lesions located on the feet were also observed, due to a significant percentage of ALM.

Studies similar to ours, with well-documented cohorts, are of great importance for better comprehension of cutaneous melanoma, and also for guiding physicians regarding how to manage such patients and conduct prevention campaigns among the population.

## References

[1] Parker SL, Tong T, Bolden S, Wingo PA (1996). Cancer statistics, 1996. CA Cancer J Clin.

[2] Greene FL, Page DL, Fleming ID (2002). AJCC Cancer Staging Manual.

[3] Mikkilineni R, Weinstock MA, Sober A, Haluska F (2001). Epidemiology. Skin cancer.

[4] Bakos L (1991). Melanomas malignos e etnia. [Malignant melanoma and race]. An Bras Dermatol.

[5] Lucas EA, Deps PD, Lima JGB, Toribio R, Gomes CC (1994). Melanoma maligno: estudo casuístico retrospectivo de 1982 a 1992, no Hospital Universitário da UFES. [Malignant melanoma: retrospective study in the period 1982-1992, in the University Hospital of UFES]. Arq Bras Med.

[6] Fernandes NC, Cardoso ICL, Maceira J, Perez M (1996). Melanoma: estudo retrospectivo de 47 casos. An Bras Dermatol.

[7] Giavina-Bianchi MH, Festa Neto C, Sanches Jr JA (1996). Análise de 115 casos de melanoma. Jornal Dermatológico da Sociedade Brasileira de Dermatologia (SBD).

[8] Bernardi CDV, Favaretto AL, Brancher MC, Ponzio HA (1998). Freqüência de melanoma maligno no Serviço de Dermatologia da ISCMPA/UFRGS. [Rate of malignant melanoma in Service of Dermatology of ISCMPA/UFRGS]. An Bras Dermatol.

[9] Criado PR, Vasconcellos C, Sittart JAS (1999). Melanoma maligno cutâneo primário: estudo retrospectivo de 1963 a 1997 no Hospital do Servidor Público Estadual de São Paulo. [Primary cutaneous malignant melanoma occurrence diagnosed at Hospital do Servidor Público Estadual de São Paulo between the period from 1963 through 1997]. Rev Assoc Med Bras (1992).

[10] Bakos L, Wagner M, Bakos RM (2002). Sunburn, sunscreens and phenotypes: some risk factors for cutaneous melanoma in southern Brazil. Int J Dermatol.

[11] Gon AS, Minelli L, Guembarovski AL (2001). Melanoma cutâneo primário em Londrina. [Primary cutaneous melanoma in Londrina]. An Bras Dermatol.

[12] Lapa MS, Guedes KF, Schalch FO, Landman G (2002). Melanomas malignos cutâneos tratados no Hospital do Câncer de São Paulo: Estudo retrospectivo para avaliação de distribuição, fatores prognósticos e sobrevida. [Cutaneous malignant melanomas treated at the Hospital do Cancer in São Paulo: Retrospective study for the evaluation of distribution, prognostic factors and survival]. An Bras Dermatol.

[13] Pinheiro AMCF, Horácio C, Rodrigues ALSV, Abe H (2003). Melanoma cutâneo: características clínicas, epidemiológicas e histopatológicas no Hospital Universitário de Brasília entre janeiro de 1994 e abril de 1999. [Cutaneous melanoma: clinical, epidemiological and histopathological characteristics at the University Hospital of Brasília between January 1994 and April 1999]. An Bras Dermatol.

[14] Moreno M (2005). Perfil dos pacientes com melanoma cutâneo no oeste de Santa Catarina, Brasil. Grupo Brasileiro de Melanoma. Boletim Informativo do GBM.

[15] Marrett LD, Nguyen HL, Armstrong BK (2001). Trends in the incidence of cutaneous malignant melanoma in New South Wales, 1983-1996. Int J Cancer.

[16] Jemal A, Devesa SS, Hartge P, Tucker MA (2001). Recent trends in cutaneous melanoma incidence among whites in the United States. J Natl Cancer Inst.

[17] Instituto Brasileiro de Geografia e Estatística (IBGE) Censo 2000. [Internet]. http://www.ibge.gov.br/home/estatistica/populacao/censo2000/tabelabrasil111.shtm.

[18] Weinstock MA, Berwick M, Balch CM, Houghton AN, Sober AJ, Soong S (2003). Epidemiology: Current Trends. Cutaneous melanoma.

[19] National Program of Cancer Registries (NPCR) United States Cancer Statistics (USCS). [Year: 2002. Display: By Cancer Type & Race (crude)] Crude Invasive Cancer Incidence Rates and 95% Confidence Intervals by Primary Site and Race and Ethnicity, United States (Table 1.1.2.1M).

[20] Australian Institute of Health and Welfare Cancer in Australia 1997: Incidence and mortality data for 1997 and selected data for 1998 and 1999.

[21] Venegas LFP, Flores C, Blacher GG, Daudt AW, Cerski CTS (1992). Melanoma maligno cutâneo no Rio Grande do Sul: estudo de 101 casos. [Cutaneous malignant melanoma in Rio Grande do Sul, Brazil: study of 101 cases]. Rev Assoc Med Bras (1992).

[22] Bandiera DC, Prudente A, Roxo Nobre MO, Junqueira ACC (1967). Melanoma maligno. Cancerologia prática.

[23] Donato CA, Yokomizo V, Peres Rosa I (1997). Melanoma maligno: avaliação de 58 casos em um período de 11 anos (jan/85 - dez/95). Med Cutan Ibero Lat Am.

[24] Brandão M, Filardi F, Domenech J (1998). Melanoma cutâneo. Grupo Brasileiro de Melanoma. Boletim Informativo do GBM.

[25] Minelli L, Pereira VL (1983). Melanoma - estudo casuístico do Instituto do Câncer de Londrina. [Melanoma: case study at the Instituto de Cancer de Londrina]. An Bras Dermatol.

[26] Reintgen DS, McCarty KM, Cox E, Seigler HF (1982). Malignant melanoma in black American and white American populations. A comparative review. JAMA.

[27] Seiji M, Takahashi M (1982). Acral melanoma in Japan. Hum Pathol.

[28] Chang AE, Karnell LH, Menck HR (1998). The National Cancer Data Base report on cutaneous and noncutaneous melanoma: a summary of 84,836 cases from the past decade. The American College of Surgeons Commission on Cancer and the American Cancer Society. Cancer.

[29] Sober AJ, Tsao H, Balch CM, Houghton AN, Sober AJ, Soong S (2003). Acquired precursor lesions and markers of increased risk for cutaneous melanoma. Cutaneous melanoma.

[30] Dennis LK (1999). Analysis of the melanoma epidemic, both apparent and real: data from 1973 through 1994 surveillance, epidemiology, and end results, program registry. Arch Dermatol.

[31] Balch CM, Soong SJ, Gershenwald JE (2001). Prognostic factors analysis of 17,600 melanoma patients: validation of the American Joint Committee on Cancer melanoma staging system. J Clin Oncol.

